# Traumatic Brain Injury Pathophysiology and Treatments: Early, Intermediate, and Late Phases Post-Injury

**DOI:** 10.3390/ijms15010309

**Published:** 2013-12-30

**Authors:** Hanna Algattas, Jason H. Huang

**Affiliations:** 1 School of Medicine and Dentistry, University of Rochester Medical Center, 601 Elmwood Ave, Box 441, Rochester, NY 14642, USA; 2 School of Medicine and Dentistry, University of Rochester Medical Center, 601 Elmwood Ave, Box 670, Rochester, NY 14642, USA; E-Mail: jason_huang@urmc.rochester.edu

**Keywords:** Traumatic Brain Injury (TBI), inflammation, seizure, excitotoxicity, treatment

## Abstract

Traumatic Brain Injury (TBI) affects a large proportion and extensive array of individuals in the population. While precise pathological mechanisms are lacking, the growing base of knowledge concerning TBI has put increased emphasis on its understanding and treatment. Most treatments of TBI are aimed at ameliorating secondary insults arising from the injury; these insults can be characterized with respect to time post-injury, including early, intermediate, and late pathological changes. Early pathological responses are due to energy depletion and cell death secondary to excitotoxicity, the intermediate phase is characterized by neuroinflammation and the late stage by increased susceptibility to seizures and epilepsy. Current treatments of TBI have been tailored to these distinct pathological stages with some overlap. Many prophylactic, pharmacologic, and surgical treatments are used post-TBI to halt the progression of these pathologic reactions. In the present review, we discuss the mechanisms of the pathological hallmarks of TBI and both current and novel treatments which target the respective pathways.

## 1. Introduction

Individuals of all ages, background, and health status are susceptible to traumatic brain injury (TBI). Every year in the United States 1.7 million people suffer TBI and TBI is listed as a contributing cause in approximately one third of injury-related deaths [1]. While the numbers suggest a grim state concerning TBI treatment there have been improvements in its management. Over the past 30 years, deaths from severe TBI have reduced from 50% to fewer than 25% [2]. Evidence-based guidelines for TBI management were introduced in 1995 because of varied treatment approaches but in the years following there have still been lapses in consistent implementation [3,4]. One problem in the development of reliable guidelines for treatment of TBI is the varied pathophysiology of injury. TBI may be penetrating or non-penetrating, diffuse or focal, vary in severity, location, and patient characteristics, just to name a few. Additionally, since TBI is often accident-related, there are limited primary prophylactic measures. Much of the resultant acute and chronic harm from TBI is related to secondary generation of tissue damage and inflammation.

In the present review, we will attempt to describe the pathophysiology of three distinct yet over-lapping states post-injury, the early, immediate, and late phases. The early phase of damage usually occurs within 24 h of injury and is directly related to tissue damage and deregulated physiological functions, the intermediate phase takes place in the days following TBI and entails neuroinflammation, and the late phase is primarily associated with seizures and epileptogenesis and arises days to weeks after TBI. Following each phase we will describe current and novel treatments and interventions that directly target the pathophysiology of each phase. There is a wealth of TBI data with countless views on injury mechanisms and treatment modalities; thus, this review provides a detailed but limited glimpse into components of the literature.

## 2. Early Phase

Different forms of mechanical insult ensue depending on the type of TBI, including acceleration-deceleration shearing and penetrating injury. Regardless, early damage following TBI often stems from the ischemic cascade. There is a fine interplay regarding normal energy processes and disruption of this intricate path leads to decreased glucose utilization, lactic acid accumulation, reduced ATP and activity of ATP-reliant ion pumps, Ca^2+^-induced depolarization, excitotoxicity, and cellular death. The sequential ischemic cascade begins with interruption of normal blood flow and numerous experimental studies demonstrate this effect. As expected, rodents subject to penetrating ballistic-like brain injury (PBBI) display a 70% reduction in regional cerebral blood flow (CBF) ipsilateral to the injury as compared to baseline. PBBI also decreases brain tissue oxygenation tension and causes spreading cortical depolarizations shortly after injury [5]. CBF reductions are more pronounced in older compared to younger rodents, as seen with fluid percussion injury (FPI) TBI [6]. As mentioned earlier, there is overlap between pathological phases. For instance, clinical evidence has demonstrated chronic CBF reduction in particular brain regions of TBI patients which cause a lasting effect to normal functioning [7]. Indeed a small study with xenon computed tomography (CT) found CBF measurement within the first 12 h post-TBI to predict six month Glasgow Outcome Scale (GOS) values, although the authors insist a larger RCT trial is warranted [8]. Overall, the literature suggests CBF disturbance is one of the first pathological steps and the effect varies with age and time.

Compared to the rest of the body, the brain displays an extremely tight autoregulation of blood flow and any perturbance alters the brain’s normal metabolic landscape. In another PBBI model, injury reduced brain oxygen tension by 40% in the area encompassing the lesion compared to sham rats, and oxygen tension was positively correlated with fraction of inspired oxygen in the air, ranging from 21%–35% [9]. Oddo *et al*. (2012) examined brain oxygen tension in anemia patients who suffered TBI. Those anemic patients with compromised brain oxygen tension were over six times more likely to suffer unfavorable outcomes, regardless of the injury severity; suggesting proper oxygenation minimizes damage due to injury [10]. The data may be extrapolated into the clinical realm where they become especially relevant. Guidelines from the Brain Trauma Foundation promote the use of intracranial pressure (ICP) and cerebral perfusion pressure (CPP) monitoring techniques when managing TBI patients [11]. Yet, Eriksson *et al*. (2012) was quick to reveal data suggesting that ICP and CPP pressure monitoring should not be substituted for true measures of brain tissue oxygenation because oxygenation is an independent value [12].

Reduced blood flow and oxygen metabolism in the brain promotes a metabolic switch from the usual aerobic process to an anaerobic program. Lactate is a marker of anaerobic respiration and builds up in tissue deprived of oxygen. Many studies have used measures of glucose consumption or oxygen levels prove there is a reduction in normal cerebral metabolism [13,14]. Even if other vital measures are controlled, metabolic deregulation still occurs. For instance, among 76 successfully resuscitated TBI patients with managed ICP, 76% had decreased glucose and 93% had an elevated lactate/pyruvate ratio [15]. Not only might cerebral blood flow and oxygen affect metabolic functioning but also the ability for glucose to enter the brain. One study using positron emission tomography (PET) with radioactively tagged glucose demonstrated diminished uptake of glucose into both cerebral hemispheres after FPI; further, glial activation and axonal damage seemed to persist in regions deprived of glucose uptake [16]. In later phases of TBI pathophysiology, large variations in glucose levels have been associated with worse long-term outcomes, suggesting a more complicated metabolic relationship [17]. Interestingly, glucose administration after controlled-cortical impact (CCI) is neuroprotective in the hippocampus and cortex, suggesting exogenous glucose supplementation is beneficial post-TBI [18]. On the other hand, lactate is found elevated in microdialysates of patients with acute TBI but lactate nor lactate/pyruvate ratio seemed to be associated with brain hypoxia [19]. Similar results have been seen in severe TBI, suggesting lactate’s increased post-TBI is not due to ischemic factors [20]. The variation in the literature paint a confusing metabolic landscape which likely varies based on the heterogeneity of TBI and time period of analysis.

Deregulated cerebral metabolism and the favored breakdown of lactate rather than glucose necessarily lead to a deficit in cerebral energy production [21]. Subsequently, reductions in ATP lead to the failure of ATP-dependent ion channels and proteins [22]. Ischemia, reduced CBF, and altered metabolic function ultimately lead to excitotoxicity-mediated cell death, including both apoptosis and necrosis [23,24]. Early research on a cohort of TBI patients identified elevated excitatory amino acids in microdialysates of patients, at levels 50 times normal in approximately 30% of the patients; correlations between excitatory amino acid quantity and secondary brain damage was also noted [25]. Glutamate is the prime excitatory amino acid and is released via pre-synaptic vesicles or leaks out of damaged membranes after TBI. Glutamate elevates because of Ca^2+^-mediated release and decreased glial glutamate uptake [26]. Such glutamate release also correlates with age, being elevated in microdialysates of elderly TBI patients compared to their younger TBI counterparts; in the same study, other measures such as some cytokines had no quantitative change [27].

Studies *in vitro* confirmed elevated glutamate activity leads to hyperexcitability and neuronal death in a dose-response relationship [28]. Mechanistically, excess glutamate binds the NMDA receptor and promotes a massive influx of Ca^2+^ and Na^+^ leading to activation of a number of enzymes responsible for ensuing cellular damage; astrocytes are prone to excitotoxicity-mediated cell death as well [29]. Indeed, administration of amantadine, an NMDA receptor antagonist, to FPI rats improved performance in the Morris Water Maze (MWM) and promoted neuronal survival in CA2/CA3 pyramidal neurons of the hippocampus [30]. These findings are in agreement with other research where MK-801, an NMDA receptor antagonist, decreased neuronal caspase-3 expression, neuronal nitric oxide synthase (nNOS) positive neurons, and mitochondria degeneration [31]. The synthesis of nitric oxide (NO) relies on Ca^2+^ to an extent and its upregulation can lead to significant oxidative damage post-TBI [32]. Recent evidence suggests metabolites of NO could be reliable markers for severe TBI [33]. NO is a direct component of the neuroinflammatory cascade, intriguingly glutamate indirectly promotes inflammatory processes as well. Dai *et al*. (2010) demonstrated that adequately high concentration of glutamate switched the effect of the adenosine-adenosine A(2A) receptor from anti-inflammatory to pro-inflammatory [34]. Overall, it is well accepted that glutamate opens the proverbial flood gates of the cell which produce significant cellular harm.

Intracellular accumulation of Ca^2+^ due to glutamate excitotoxicity perturbs intracellular ionic concentrations and warrants mitochondria to sequester such elevated Ca^2+^ stores [35]. Isolated mitochondria from CCI experimental models demonstrate increased Ca^2+^ stores and impaired oxidative phosphorylation, another process causing metabolic deregulation post-TBI [36]. Influx of Ca^2+^ into mitochondria promotes production of reactive oxygen species (ROS) which cause additional damage at elevated levels [37]. Structurally, mitochondria exhibit swelling due to a mitochondrial permeability transition pore that compromises their function. Experimental findings demonstrated mitochondrial pathology precedes neuronal loss and can be seen as early as 30 min post-TBI in CCI rats [38]. Bouts of Ca^2+^ stress to mitochondria lead to release of cytochrome c from mitochondrial membranes and the activation of caspase, a protein involved in cell death pathways. Of clinical relevance, both cytochrome c and caspase have been identified in the CSF of patients with severe TBI [39]. Clinical evidence corroborates the importance of mitochondrial pathology since *N*-acetylaspartate, a surrogate of mitochondrial function, is correlated with TBI patient outcomes [40]. Overall, immediate physical and structural damage from TBI interrupts blood flow and oxygenation to the brain which are both tightly regulated variables. Mechanical stress and ischemia help advance the excitotoxic cascade and deregulate cerebral metabolism, producing the earliest pathological indications of TBI.

## 3. Prophylactic Hypothermia and Hyperbaric Oxygen Therapy (HBOT)

Initial management of the TBI patient is generally centered on prophylaxis and supportive measures, including blood pressure and oxygenation monitoring, infection and deep vein thrombosis prophylaxis, analgesia, and setting thresholds on vital values including ICP and CPP. Deregulation of cerebral metabolism, blood flow, and lost perfusion are early changes post-TBI. Prophylactic hypothermia is one option that directly combats the problematic nature of early TBI pathology. Hypothermia lowers cerebral metabolic rates and slows damage occurring post-TBI. For every degree Celsius decrease in temperature, brain oxygen consumption drops 5%–7%; this is capable of decreasing brain energy expenditure while maintaining blood oxygenation levels, therefore matching cerebral metabolism with the reduced cerebral blood flow [41]. Hypothermia also dampens the innate immune response post-TBI in experimental models, also demonstrating the overlap with inflammatory phase which is yet to be discussed [42]. The Brain Trauma Foundation (BTF) guidelines for severe TBI treatment posit as level III evidence that hypothermia patients do not exhibit decreased mortality compared to normothermic controls. Simultaneously, the BTF reports preliminary evidence which suggests a decrease in mortality upon maintaining target temperatures for 48 h and that patients receiving prophylactic hypothermia had higher Glasgow Outcome Scale (GOS) scores compared to normothermic patients [43].

Prophylactic hypothermia has received mixed results in the literature because of multiple variables involved in its successful implementation; these include temperature at time of injury, initial onset of cooling, rate of cooling, final temperature sought, and mechanism of cooling. Early research corroborated such variability by finding spontaneous hypothermia upon time of admission to be associated with poorer prognosis [44–46]. Thus, it may be said that hypothermia administration is as heterogeneous as the TBI pathology itself. Since brain temperature cannot be predicted with high confidence from body temperatures separate monitoring is recommended [47]. The National Acute Brain Injury Study: Hypothermia II was a large scale RCT which failed to confirm any benefit of hypothermia [48]. However, recent retrospective analysis of pooled neurotrauma data revealed patients receiving hypothermia treatment had significantly higher favorable outcomes compared to normothermic patients and those with no temperature management; it is important to note that hypothermic patients were, on average, significantly younger [49]. Indeed, other studies have replicated these findings. One such study demonstrated hypothermia of 32.7 °C for 72 h produced favorable outcomes [50]. The same study also identified hyperglycemia to be an independent risk for poor outcome and that hypothermic patients had reduced glucose concentrations compared to normothermic counterparts. Such a finding suggests hypothermia may promote favorable outcomes by decreased glucose levels. A meta-analysis by Fox *et al*. (2010) suggests a rationale for discrepancies among the literature. That group found hypothermia studies with long-term/goal-directed strategies in their design concluded patients to have lower mortality and more favorable outcome whereas studies implementing short-term strategies were often inconclusive (Table 1) [51]. Besides strategic design, another key variable influencing prophylactic hypothermia studies is re-warming strategy. For instance, the National Acute Brain Injury Study: Hypothermia II re-warmed patients from 33 °C by 0.5 °C every two hours which found no difference in GOS or mortality between hypothermic and normothermic patients [48]. Another prospective study cooled patients to 32.7 °C and allowed them to spontaneously re-warm at room temperature. Importantly, that study found hypothermic patients to have significantly improved GOS compared to the normothermic group [50]. Undoubtedly then, re-warming strategies may be just as significant as cooling ones in optimizing patient outcomes.

Pairing hypothermia with other treatment strategies may accentuate benefits. For example, concurrent monitoring of brain tissue oxygen and administration of mild hypothermia synergistically assists in reducing ICP post-TBI; decreasing ICP may limit inflammatory damage occurring later in TBI pathogenesis [52]. Identifying cases of TBI where the use of hypothermia is warranted is vital. In agreement with previously mentioned data, xenon- and perfusion-CT analyses demonstrated disturbances in cerebral blood flow (CBF) in TBI patients [53]. In that study, Honda *et al*. (2013) found CBF in focal TBI patients to be more perturbed than in diffuse TBI [53]. The research group suggested moderate hypothermia should be used in managing cases of TBI involving larger CBF changes. Of extreme clinical importance, data from pediatric TBI patients demonstrated that phenytoin elimination is decreased after hypothermia administration, and this is especially important because phenytoin is a recommended anti-epileptic used in the treatment of acute post-traumatic seizures and has non-linear metabolic characteristics [54]. Ultimately, the potential for widespread use of hypothermia is possible but requires more research. Trials to continue examination of hypothermia’s therapeutic potential are underway, including a multi-center trial by Andrews *et al*. (2013) and the POLAR-RCT in Australia and New Zealand [55].

Hyperbaric oxygen therapy (HBOT) is another intervention used in early prophylactic treatment of TBI. HBOT encompasses the inhalation of 100% oxygen at environmental pressures above one atmosphere. As spoken to earlier, deregulation of CBF produces an oxygen deficit causing metabolic modifications and ischemia. By increasing the partial pressure of oxygen in blood, independent of that bound to hemoglobin in erythrocytes, HBOT increases oxygen saturation reaching the brain and attempts to decrease tissue damage secondary to ischemia and hypoxia [56]. Yet, since most O_2_ is hemoglobin-bound, HBOT-mediated O_2_ saturation increase is limited to up to 10%; a clinically significant amount in many cases. Small scale, early research proved treatment with 100% oxygen for six hours reduced lactate and increased brain tissue oxygenation [57]. More extensive evidence from an early systematic review deemed HBOT’s therapeutic benefit inconclusive [58]. Yet, a recent retrospective study found TBI patients treated with HBOT have improved outcomes when compared to control counterparts [59]. Additionally, prospective studies administering HBOT after patients’ conditions stabilized also demonstrated improved outcomes based on GCS and GOS [60]. One large clinical trial examined the efficacy of HBOT followed by normobaric hyperoxia treatment (NBH) for three days and found the treatment group had reductions in ICP, mortality, and cerebral toxicity with improved favorable GOS outcomes [61]. In an earlier study, Rockswold *et al*. (2010) compared HBOT to NBH and demonstrated the effects of both to be very similar compared to a standard care control group (Table 2) [62].

Variations in HBOT administration and TBI circumstances may alter outcomes. In mild TBI, HBOT did not significantly differ from a sham treatment when analyzing post-concussive symptoms, suggesting an injury severity interaction [63]. Still, the study by Wolf *et al*. (2012) used 2.4 ATA HBOT whereas 1.5 ATA HBOT in chronic blast-induced mild to moderate military TBI patients improved symptoms and cognitive outcomes, suggesting an administration interaction [64]. With mounting evidence, HBOT may become a treatment for targeted types of TBI. For instance, regional CBF measurements in healthy controls provided HBOT revealed that blood flow was increased to specific areas, including cerebellum, sensory-motor, premotor, visual, and posterior cingulate cortices compared to normoxic patients. Interestingly, normoxic patients had increased CBF to many subcortical structures, including the hippocampus [65]. Thus, the data suggest identifying regions of deregulated CBF before administering HBOT may be beneficial. Lastly, it is essential to be mindful of the adverse effects of hyperoxic treatments. For instance, hyperoxia in healthy patients has been shown to produce a small decrease in CBF owing to vasoconstriction of vasculature [66]. Experimental studies suggest hyperoxic treatments may increase free radical oxygen species generation [67]. However, the findings have been double-edged; Puccio *et al*. (2009) did not find significantly altered oxidative stress markers in the CSF of TBI patients after a two hour stint of normobaric hyperoxia [68]. Overall, the present evidence provides conclusions on both sides of the spectrum regarding HBOT. Indeed, understanding the pathophysiology of TBI and the specific patient’s case provides insight into when HBOT may be clinically indicated.

## 4. Intermediate Phase

The brain is considered an “immune-privileged” organ due to the presence of the selective blood-brain barrier (BBB) that impedes the entry of many foreign pathogens and immune mediators. However, TBI compromises this blockade and allows entry of chemical messengers and immune cells into the brain parenchyma; furthermore, TBI impacts central cytokine release within the brain itself. There are numerous postulated triggers of post-TBI inflammation, including: peripheral blood products, tissue and cellular debris, complement fragments, prostaglandins, and reactive oxygen and nitrogen species (RNS) [69]. Inflammation produces two disparate effects on brain tissue, on one end causing damage and the other promoting regeneration. For instance, activation of microglia promotes recovery via phagocytosis of debris; however, excessive cytokine and chemokine secretion prolong the inflammatory process. The inciting event to the inflammatory progression is the mechanical damage brought on by neurotrauma, causing the release of the aforementioned triggers and evoking a series of cellular events culminating in inflammation. A main measurable pathological sequela of neuroinflammation is elevated ICP which many of the treatment modalities to be discussed target.

After the initiating injury, up-regulation of central nervous system (CNS) chemokines does not occur immediately. For instance CCL20, a lymphocyte chemotactic, increased centrally 48 h post-injury in a lateral FPI rat model of TBI. Interestingly, expression of CCL20 was increased in the periphery 24 h post-injury, suggesting a peripheral response prior to a central response [70]. Microglia, astrocytes, and neurons are all capable of producing additional chemokines in response to local inflammation [71]. The varying cell types have different responses to chemokines. The chemokine inflammatory proteins CCL3 and CXCL2 increase in mice post trauma, yet the MIP-2 receptor, CXCR2, increased expression only on astrocytes [72]. This data suggests targeted activity towards certain types of glia. Secreted chemokines encourage the expression of adhesion molecules on blood vessels which allows leukocyte extravasation from the periphery into the brain parenchyma. Leukocyte and lymphocyte entry into the CNS continues the inflammatory progression, though. However, such an infiltrate is time dependent because extravasation occurs slowly; neutrophil levels peak approximately two days post-TBI and monocytes slightly later [73]. Yet, a blast wave-induced TBI rat model found polymorphonuclear (PMN) leukocytes and lymphocytes in brain parenchyma within one hour of injury [74]. Regardless, neutrophils are the primary leukocytic infiltrate in the plasma of TBI patients acutely after injury [75]. Leukocyte homogenates from post-TBI patients display up-regulation of inducible-nitric oxide synthase (iNOS), cyclooxygenase-2 (COX-2), and nicotinamide adenine dinucleotide phosphate-oxidase (NADPH oxidase); all enzymes involved in producing the damaging neutrophilic oxidative burst and. Indeed, flow cytometry has confirmed increased oxidative activity in that leukocyte population [75].

Post-TBI there is increased neutrophilic infiltration, astrocytosis, edema, and both pro- and anti-inflammatory cytokines. The major pro-inflammatory cytokines released are interleukin-1β (IL-1β), interleukin-6 (IL-6), and tumor necrosis factor alpha (TNFα). The anti-inflammatory cytokines are interleukin-10 (IL-10) and transforming growth factor beta (TGFβ) [76]. Other significant cytokines and chemokines involved in the pathophysiology of TBI have been reviewed elsewhere by Ziebell and Morganti-Kossman (2010) [77]. IL-1β and TNF are found present one hour post-injury, remain elevated for three weeks, and are accompanied by astrocytosis [74]. IL-1β is an especially potent pro-inflammatory cytokine capable of driving most of the inflammatory processes seen post-TBI. IL-1β is secreted by immune cell mediators and its processing is promoted by production of the NLRP3 inflammasome post-TBI [78]. Frugier *et al*. (2010) found IL-1β mRNA and that of IL-6, IL-8, and to be elevated *in situ* in post-mortem human brains after acute cerebral injury; interestingly, anti-inflammatory cytokine protein levels were unchanged [79]. IL-1β spurs a positive feedback mechanism leading to activation of microglia and further pro-inflammatory cytokine release. Microglia stimulated with IL-1β are activated and express the Krüppel-like factor 4 (Klf4) via a PI3K/Akt pathway leading to further production of IL-1β. Additionally, there is increased COX-2, monocyte chemoattractant protein-1, and IL-6, as well as decreased expression of iNOS [80]. Importantly, IL-1β acts uniquely on astrocytes. When astrocytes are damaged as occurs in TBI, IL-1β activates the intracellular ERK pathway which releases matrix metalloproteinase-9 (MMP-9) from astrocytes *in vitro* [81]. MMPs degrades extracellular matrix and further promotes BBB breakdown promoting and prolonging neuroinflammation. The levels of MMP-9 and MMP-8 correlate with interleukin and TNFα levels in microdialysate and CSF of patients after severe TBI [82].

Secretion of TNFα by astrocytes and microglia occurs rapidly after TBI, with mRNA and protein levels detectable within 17 min of injury as measured in post-mortem brains of patients who died shortly after TBI [79]. TNFα action in the chronic neuroinflammation setting produces spatial learning and memory deficits, yet treatment with a TNFα protein synthesis inhibitor, 3,6′-dithiothalidomide (DT), actually spares these cognitive effects [83]. Interestingly, DT treatment spares IL-1β levels and still rescues behavioral function, suggesting TNFα plays a larger role in cognitive dysfunction. The detrimental effects of TNFα have been validated in clinical studies also. A cohort of 1096 TBI patients was analyzed for the effect of cytokine gene polymorphisms on Glasgow outcome scores. Homozygous carriers of the TNFα-308 single nucleotide polymorphisms (SNP) had significantly worse outcomes after TBI; the SNP is present in the TNFα promoter and is linked to elevated TNFα levels [84,85]. In mice TNFα signaling via TNFR1 is positively linked to the expression of a myriad of gene products including aquaporin-4 (AQP4), a water channel which influences edema formation and is predominantly expressed in astrocytes; yet, this up-regulatory effect is not seen in cultured astrocytes [86,87].

Edematous changes during the inflammatory phase of TBI are tightly linked to regulation of water and ionic flow between the extracellular fluid and glia. As mentioned, AQP4 is a mediator of water homeostasis and appears to be the predominant AQP responsible for edema formation post-TBI [88]. AQP4 is upregulated in rat cortex as soon as 3 and 48 h post-injury in a blast-induced moderate neurotrauma experimental model; the same study also saw TNFα, C3/C5b-9, and leukocyte infiltration all increase at those time points [89]. Indeed, AQP4 is also elevated in the CSF of severe TBI patients compared to healthy controls [90]. Brain edema which results from AQP4 over-expression is associated with increased fluorojade immunostains (marker of degenerating neurons) and neurobehavioral deficits [91]. Nevertheless, AQP4 also has advantageous effects in TBI pathophysiology. AQP4 has been shown to promote astrocytic scar formation and reduce post-TBI seizure severity; further establishing that inflammation during TBI has detrimental and beneficial effects [92]. Evidence also suggests the edematous response post-TBI is age dependent. Two month old and 21 month old rats subjected to CCI displayed similar morphological damage, yet the older rats had increased edema and more rapid onset of poor neurological outcome compared to those younger [93]. Edema directly contributes to the previously mentioned hallmark of TBI, raised intracranial pressure (ICP). Raised ICP in pediatric TBI patients produces long term detrimental abnormalities in cerebral architecture. Tasker *et al*. (2010) followed these pediatric patients for 4.9 years post-TBI and revealed decreased cross-sectional area, increased compaction, and thinning of the corpus callosum in addition to reduced fractional anisotropy [94].

As has been detailed, a number of cytokines, chemokines, and protein molecules enhance the inflammatory response post-TBI. Lloyd *et al*. (2008) attempted to reduce not one but many of these inflammatory mediators to understand their collective efforts. Mice treated with Minozac, an experimental therapeutic, after CCI had attenuated pro-inflammatory cytokines, less astrocyte activation, and no increase in brain edema [95]. This experimental data suggests global dampening of the inflammatory process improves outcomes. Minozac is but one pharmacological treatment studied in experimental models of TBI, numerous others have been reviewed elsewhere [96]. It then logically follows that quantifying the state of inflammation post-TBI would be therapeutically beneficial. Biomarkers of inflammation may serve as a quantitative figure to assess TBI severity. S100 calcium binding protein B (S100B) is secreted by astrocytes into the CSF upon injury and displays a strong correlation with injury severity as measured in head trauma patients. S100B, however, does not readily cross the BBB and also increases in response to peripheral trauma, making its use less feasible [97]. Hernanadez-Ontiveros *et al*. (2013) suggest the use of activated microglia as a TBI marker and useful criteria to influence therapeutic interventions; as is made explicit this task relies on decoding the unique cytokine and chemokine profile for such microglia after TBI [98]. Understanding the inflammatory cascade and its variability within different forms of TBI will be paramount in effectively treating subsets of injury.

## 5. Progesterone, Hyperosmolar Agents, Decompressive Craniectomy (DC)

There are no true guidelines for the treatment of the neuroinflammatory phase of TBI, but rather monitoring technologies and secondary prevention tactics aimed at ameliorating its sequelae, namely elevated ICP. ICP monitoring is recommended as level II evidence when GCS falls between 3 and 8 and is accompanied by an abnormal CT scan [99]. One strong contraindication for use in reducing ICP is corticosteroid administration, namely methylprednisolone. The widely acclaimed CRASH trial found elevated risk of death in patients administered methylprednisolone after brain injury [100]. Current research has explored other options including pharmacotherapies and surgical options, both of which will be discussed in light of ICP.

Progesterone, an endogenous steroid hormone, is a pharmacotherapy option gaining recent attention. Attella *et al*. (1987), somewhat serendipitously, first revealed the neuroprotective effects of progesterone. His experiments demonstrated reduced edema in pseudopregnant rats after frontal cortex lesions when compared to normal cycling rats [101]. Progesterone acts on the membrane bound progesterone receptor (mPRα) which is expressed in neurons but not glia in the mouse brain. Strikingly, upon induction of TBI, mPRα increases expression on oligodendrocytes, astrocytes, and reactive microglia, implying a role of progesterone in neuroprotection [102]. Since the early experiments others have explored progesterone’s neuroprotective effect. For instance, Wright *et al*. (2001) found TBI-rodents administered progesterone had significantly reduced cerebral edema compared to controls [103]. Progesterone may reduce cerebral edema via elevation of *P*-glycoprotein expression, a marker tightly linked to BBB function [104]. In addition, progesterone modulates AQP4 expression spatially and temporally after TBI, affecting edema formation [105]. Trauma often leads to vascular injuries as well, and, appropriately, progesterone has been found to increase circulating endothelial progenitor cells post-TBI, suggesting a role in vascular remodeling [106]. The deleterious pro-inflammatory cytokines IL-6 and TNFα, pro-apoptotic caspase-3 and bax, and the marker of lipid peroxidation 8-isoPGF2 have all been proven to be reduced by progesterone in rodent models of TBI [104,107–112]. Such cytokines and stressors lead to cell death in susceptible regions of the brain, notably the DG of the hippocampus. Yet, progesterone also reduces cell death in the DG of rats post-TBI [113]. Of note, progesterone administered with vitamin D after TBI reduces astrocyte proliferation and neuronal loss with a trend toward improved memory outcomes post-TBI when compared to rats supplemented with progesterone alone [114].

Besides experimental data, progesterone has garnered attention in clinical trials. The ProTECT RCT was one of the first to examine progesterone’s efficacy post-TBI, reporting no serious adverse events and a lowered thirty day mortality rate compared to placebo [115]. A more recent study found five days of progesterone administration to TBI patients with a GCS less than or equal to 8 led to marked improvement at three month follow-up compared to placebo patients [116]. A pooled meta-analysis of small progesterone RCTs revealed progesterone reduces risk of mortality (RR = 0.61) and had a lower risk of death or severe disability (RR = 0.77) [117]. As alluded to earlier, rodent studies have suggested vitamin D together with progesterone has synergistic benefits. In agreement, patients administered intramuscular progesterone followed by vitamin D within 8 h of TBI had elevated recovery rates, GOS outcomes, and reduced mortality [118]. More positive studies are needed to fully warrant the use of progesterone. The large scale SyNAPSe trial is one such RCT currently underway. The RCT hopes to determine if IV progesterone given with 8 h of TBI for a total of 120 h enhances patient recovery compared to placebo administered patients (Table 3).

Neuroinflammation promotes edema formation and expansion within the spatially limited cranial cavity, thus increasing ICP. Prolonged ICP elevation can be the stem from which many pathogenic features of TBI arise. The BTF currently recommends, as level II evidence, the use of mannitol for the control of raised ICP after TBI while maintaining systolic blood pressure above 90 mmHg [119]. By controlling ICP, mannitol allows diagnostic and interventional procedures, such as CT scan and intracranial evacuation, to be completed more easily. Mannitol’s effectiveness may stem from promotion of vasoconstriction, thus lowering ICP [120].

Hypertonic saline (HS) solutions are other hyperosmolar agents which have been the target of ongoing research because of fewer effects on blood pressure compared to mannitol. Both treatments reduce ICP yet a recent meta-analysis found a trend favoring hypertonic sodium solutions because of greater ICP reduction [121]. One such solution is sodium lactate which has been independently found to outperform mannitol in terms of reducing ICP (7 mmHg drop *vs*. 4 mmHG) and acting longer; sodium lactate has also been successful at more dilute (half molar) concentrations [122]. HS is also useful in cases of elevated ICP which do not respond to other therapies. For instance, repeated administration of 14.6% HS in a cohort of patients with elevated ICP completely refractory to other therapies was shown successful in reducing ICP [123]. Other studies have confirmed this finding by directly comparing mannitol and HS in similar refractory cases of elevated ICP. One such study also revealed HS to significantly elevate brain oxygenation compared to mannitol [124]. By reducing ICP, hyperosmolar agents may elevate cerebral perfusion pressure (CPP) which is beneficial when focal regions are hypoperfused from trauma; mannitol and HS both have demonstrated this effect in an eight-patient, acute TBI cohort [125]. Yet, when compared with mannitol directly in a randomized trial, HS increased cerebral blood flow (CBF) and CPP more and for an increased duration [126]. Although the data for HS is appealing the BTF does not have enough evidence to support HS over mannitol at this point in time (Table 4).

A slightly more controversial procedure used in managing raised ICP is decompressive craniectomy (DC) where a skull flap is removed to allow space for the swelling brain. Early trials revealed positive outcomes in greater than 50% of patients with severe TBI based on GOS-E [127]. However, the procedure maintains a high mortality rate of 26.4% post-operation with the rate being higher with increased age and lower GCS score [128]. Interestingly, when DC is paired with mass evacuation, mortality rate actually decreases compared to DC without mass evacuation in TBI patients [129]. In addition to mortality rate, DC is associated with numerous adverse outcomes, including: contusion expansion, new contralateral subdural or epidural hematoma, CSF leakage, epilepsy, cerebral herniation, subdural effusion, and infection. In agreement with previous studies, adverse outcomes were more common when GCS score was below 8 and age above 65 [130]. Still, blast-induced TBI occurring in the combat arena is one context where DC is aggressively used, partially because of the younger age and health status of military members [131]. Regardless of potential adverse effects, DC does successfully control ICP and CBF in TBI patients [132,133]. On the other hand, DC is associated with reduced cerebral metabolic rate of oxygen, which is a value positively correlated with functional outcomes [132]. The DECRA trial was one large scale RCT which examined the usefulness of DC after diffuse TBI. In that trial DC was effective in rapidly reducing ICP and time in the intensive care unit however it led to worse outcomes; 70% had an unfavorable outcome in the DC group compared to 51% in the standard care group [134]. The conversation regarding the DECRA trial has been ongoing due to problematic study design, causing some to dismiss its clinical influence. Honeybul *et al*. (2013) point out that patients randomized to the surgery arm of the trial may have had more severe injuries and that substantial crossover from the standard care group to the surgery group skewed the results [135]. Of note, when DC is performed up to a week after TBI there are still comparable GOS outcomes as compared to those having the procedure done within a day after injury and those receiving a standard, conservative treatment [136]. One intriguing aspect of DC is that it is very cost-effective compared to comfort care at a range of patient ages [137]. However, this cost-effectiveness wanes as the severity of TBI increases [138]. The overall picture regarding DC’s use post-TBI is summed up nicely by Lemcke *et al*. (2010), who posits that the prognosis post-operation is generally poor but predictive indicators of outcome should be taken into consideration, including age, midline shift, and quality of the basal cisterns on head CT [139].

## 6. Late Phase

Seizures are among the more prominent long-term sequelae of TBI, progressing into epilepsy in more severe cases. The initial cellular trauma brought on by TBI promotes cell death and inflammation. Intriguingly, greater and prolonged BBB disruption is seen in patients who develop post-traumatic epilepsy (PTE) compared to those who do not develop epilepsy after TBI, suggesting inflammation and infiltration play a role in epileptogenesis [140]. Nonetheless, the ultimate purpose of inflammation is to clear the damage and debris so repair may ensue. Self-repair processes modify neuronal circuitry and may lead to an epileptogenic transformation in focal or diffuse areas [141]. Seizures post-TBI are generally classified based on onset time: immediate (<24 h post-injury), early (<1 week post-injury), and late (>1 week post-injury). Early and late seizures will primarily be discussed here as they are more often due to altered neural circuitry rather than direct, immediate sequelae of injury.

In a simple sense, seizures develop when excitatory potentials become favored over inhibitory potentials and this affects the synchronous entrainment of multiple neurons. The hippocampus and cortex are especially prone to epileptiform activity. In the hippocampus of rats, TBI reduces Kv.4.2 (A-type K^+^ Channel) expression and current flow, causing neurons to become more excitable and more prone to bicuculline-induced seizures [142]. Hippocampal mossy fibers connecting granule cells of the dentate gyrus (DG) with CA3 are re-organized post-TBI, possibly involving activation of the trkB-ERK1/2-CREB/Elk-1 signaling path [143]. Data suggest trkB expression is coincident with GAP43 expression, a marker of axonal growth; extension of neuron processes alters neural circuitry and mossy fiber sprouting (MFS) is one such change [144]. MFS seems necessary for epileptiform activity. For example, brain slices from CCI mice have abnormal electrical activity, including elevated excitatory post-synaptic currents (EPSC), but those injured without MFS do not significantly differ from controls [145,146]. MFS is a consistent pathological hallmark, and is seen in the ipsilateral DG of 95% of mice 8–12 weeks after suffering a brain injury (however, sprouting may be present earlier) [146]. Such sprouting depends on the severity of TBI and whether the cortex impinges on the hippocampus [147]; for instance, 20% of mild-CCI mice displayed spontaneous seizures whereas 36% of severe-CCI mice suffered the same affliction [145]. Molecular analysis of the hippocampi of epileptic patients who suffered trauma demonstrates MFS in addition to focal cell loss in the hilar region of the DG. The patients studied had MFS which was present from 4–18 years post-injury; such data grounds MFS as clinically relevant [148]. In our lab, using a CCI mouse model, we demonstrated administration of imipramine, a tricyclic antidepressant, to stimulate hippocampal neurogenesis. Of note, most bromodeoxyuridine (BrdU)-positive progenitors became neurons in the DG and astrocytes in the hilus [149]. Since the hippocampus is particularly susceptible to damage, the stimulation of neurogenesis and reduction in cell death may be a means by which to reduce seizure activity. Our lab identified Nogo-66 receptor 1 (NgR1) to inhibit recovery post-CCI in mice; mice lacking NgR1 displayed improved performance on the Novel Object Recognition test and increases in markers of cell proliferation and recovery compared to control CCI mice [150].

Reduction of inhibitory currents is another means by which TBI promotes seizure activity. The hippocampus relies on inhibitory GABA currents for proper function and disruption leads to abnormal electrical activity. For instance, one month after FPI, rats display reductions in GABA_A_ currents in ipsilateral DG granule cells, eventually progressing to the hippocampus contralateral to injury by six months [151]. Also, bathing GABA_A_ antagonists on hippocampal slices from experimental TBI rats elicits the abnormal hyperexcitability in the granule and molecular cell layers [152]. In particular, GABAergic hippocampal interneurons of the parvalbumin, calretinin, or neuropeptide Y immunoreactive classes are affected by TBI [153]. The DG and CA3 regions are also primarily vulnerable as seen in an intricate stereological FPI rat model that induced interneuron death in the DG and CA3 with resultant increased excitability in the DG. Interestingly, however, the CA displayed reduced excitability in that study [154].

Overall, synaptic alterations in inhibitory and excitatory circuits of the hippocampus play a role in seizures post-TBI. Notably, the hippocampus may be a key biomarker in identifying susceptibility to epileptic activity. PET and MRI have been used to identify functional and structural changes post-TBI which may account for epileptic outcomes. Most areas studied could not foretell outcome, however ipsilateral hippocampus surface shapemeasured with PET and analyzed using multivariate logistic regression could predict epileptic outcomes in rats [155]. Similar results extend into the clinical realm where volumetric MRI of TBI patients demonstrated greater hippocampal atrophy in those with seizures [156]. Besides the hippocampus, the cortex is also particularly prone to epileptiform activity. One week post-injury, pyramidal cells in layer V of the neocortex of CCI-rats displayed evoked abnormal discharges followed by repetitive post-discharge; by two weeks the evoked activity progresses and spontaneous discharges also occur [157]. Similar mechanisms of epileptogenesis exist in the cortex and hippocampus yet pharmacological therapies do not make a distinction upon where they preferentially act.

## 7. Seizure Treatments

Seizures are common post-TBI occurring in approximately 50% of patients 15 years after a penetrating injury. Post-traumatic seizures may be classified as early (<1 week post-injury) or late (>1 week post-injury), with an incidence of 4%–25% and 9%–42%, respectively, in untreated patients [158–160]. There are numerous factors which put patients at increased risk for post-traumatic seizures, including: GCS < 10, cortical contusion, depressed skull fracture, subdural or epidural hematoma, intracerebral hematoma, penetrating head wounds, and seizures within 24 h of injury [161,162]. According to the Brain Trauma Foundation’s (BTF) management guidelines there are no level I recommendations for anti-seizure prophylaxis. Rather, there are level II recommendations which suggest using anticonvulsants such as phenytoin and valproate to prevent early seizures but not late seizures because of the side-effects associated with chronic use [163]. Other anti-convulsants such as phenobarbital and carbamazepine are generally avoided because of adverse effects and pharmacodynamic profile [164]. Besides acute therapy, there is a relative paucity in pharmacological options for seizure prophylaxis post-TBI; yet, promising agents have shown success in preliminary trials.

Levetiracetam (LEV) is an anticonvulsant which binds synaptic vesicle glycoprotein 2A (SV2A) and likely inhibits presynaptic Ca^2+^ channels [165,166]. In basic science work, intraperitoneal LEV given daily to rats which suffered CCI led to improved motor function, reduced hippocampal cell loss, decreased contusion volumes, and reduced IL-1β expression [167]. Clinically, a recent phase II trial among 20 paediatric cases of TBI showcased LEV as a feasible option to prevent seizures in high-risk patients because of its safety and lack of adverse events [168]. Additional studies would have to compare LEV to phenytoin (PHT), the current acute prophylactic standard. One comparison was a meta-analysis revealing equal efficacy between PHT and LEV; the authors suggested further high quality RCTs be completed before conclusions are drawn [169]. Addition of electroencephalography (EEG) to trials comparing outcome after PHT or LEV administration demonstrated that epileptiform activity and discharges were not predictive of outcome in either group [170]. Kruer *et al*. (2013) completed a retrospective observational study comparing PHT and LEV. Of 109 patients studied (89 receiving PHT and 20 with LEV), only one patient in each group suffered a post-traumatic seizure with a trend favoring LEV [171]. Interestingly, anticonvulsant therapy was continued past seven days in that study, against the present guidelines. IV administration of LEV and PHT in a prospective, randomized trial showed LEV to improve long-term outcomes based on Disability Rating Score and GOS but have no effect on seizure occurrence compared to PHT [172]. Other prospective multicenter comparisons of PHT and LEV for prophylaxis of acute seizures found no significant improvement in outcomes when LEV was administered [173]. Most studies have examined LEV *vs*. PHT in treating early but not late seizures. Recently a Phase III trial wishing to examine LEV’s ability to reduce both early seizures and late epilepsy was terminated due to small enrolment, leaving such a question yet to be answered.

The literature is in disagreement concerning the efficacy of LEV as a first-line treatment. Yet, LEV is still appealing because it does not require serum monitoring, which PHT demands owing to its non-linear metabolism. However, Pieracci *et al*. (2012) make the argument PHT is more cost-effective than LEV to both the institution and patient, ultimately recommending PHT remain the first-line therapy [174]. Cotton *et al*. (2011) quantified costs of a seven-day course of PHT and LEV, revealing the values to be 37.50 USD and 480.00 USD, respectively [175]. Still, significant disagreement regarding cost-effectiveness exists. Kazerooni *et al*. (2010) reported LEV as having the potential to be a more fiscally conservative option; based on experimental data they calculated the incremental cost-effectiveness ratio of LEV/PHT for each successful seizure prophylaxis regimen to be 360.82 USD [176]. Regardless of monetary incentive, accounting for valuable clinical time saved in forgoing serum monitoring of patients may be enough to give LEV an advantage over PHT.

As mentioned earlier, barbiturates, such as phenobarbital, are avoided as a first line therapy due to adverse effects [164]. Trials in Europe examining the effects of barbiturates on treatment were completed to no avail. While high dose barbiturates successfully decrease the elevated ICP typical of TBI they also lead to extended hemodynamic instability even when vasopressive agents are administered [177]. Valproate has been studied for early seizures with results demonstrating a trend toward favorable outcomes but the study failed to achieve significance and was not adequately powerful to detect a change [178]. Excitingly though, a phase III study sponsored by the National Institute of Neurological Disorder and Stroke evaluating valproate against PHT for seizures post-TBI was recently completed but the results not yet released. Topiramate, yet another anticonvulsant, is currently being studied in the PEPTO trial to compare it to PHT in preventing epilepsy after TBI. Recent evidence demonstrates ethosuximide, another anti-convulsant, as capable of decreasing the incidence, frequency, and delaying the onset of non-convulsive seizures in rats suffering a penetrating ballistic-like brain injury [179]. However, ethosuximide treatment has not been attempted in the clinical arena (Table 5).

A surgical option for treatment of post-traumatic epilepsy is implantation of a vagal nerve stimulator (VNS). Generally, a small stimulator is implanted in the chest wall in the vicinity of the vagus nerve; it is believed the stimulator acts by altering norepinephrine and elevating GABA levels [180]. VNS effects have been observed in rodent models of TBI but few clinical studies have been completed. Englot *et al*. (2012) retrospectively compared outcomes of VNS in patients with post-traumatic epilepsy (PTE) and those with non-traumatic epilepsy. The group found PTE patients to have larger reductions in seizure frequency and a greater clinical response compared to non-traumatic epileptics [181]. Current pilot studies by Dr. Samadani and colleagues to understand prospective clinical outcomes in patients who receive a VNS after TBI are underway and successful work would pave the way for larger trials [182].

## 8. Conclusions

**Figure 1.**
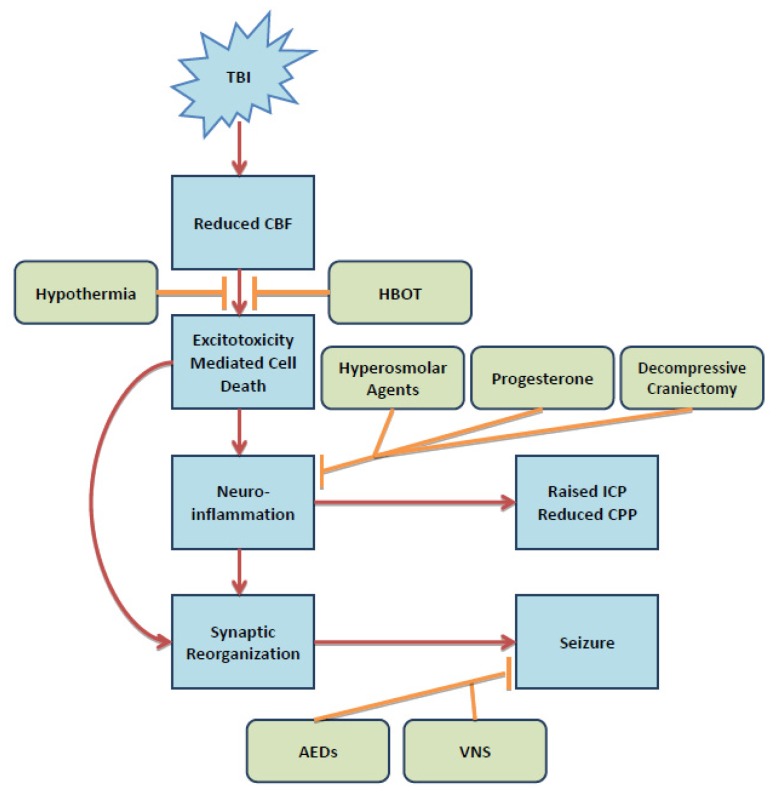
TBI pathologic process and treatment targets.

The present review outlines the pathophysiological processes which occur post-TBI and treatments aimed at ameliorating them. The distinction between phases is dependent upon the processes rather than being separated in time. Penetrating injury, mechanical stress, acceleration-deceleration injury, and shear forces provide the direct trauma-induced damage. These forces deregulate CBF and cause direct cellular-injury which leads to excitotoxic neuronal death. The reduced CBF deregulates cerebral metabolism and depletes energy stores within the brain. Prophylactic hypothermia and HBOT are two treatments which aim to reduce the energy expenditure of the brain and provide increased O_2_, respectively. Both treatments aim at pushing cerebral metabolism toward its normal, aerobic state. Elsewhere, we provide a detailed review of the technology and areas of improvement regarding both prophylactic hypothermia and HBOT [183]. Following immediate cell death and debris accumulation resident immune cells release cytokines and chemokines promoting neuroinflammation. The immediate damage resulting from trauma also comprises the BBB and allows entry of circulating immune regulators which contribute further to neuroinflammation. Numerous trials have recognized progesterone’s neuroprotective potential and ability to combat inflammatory processes. Inflammation is also characterized by cerebral edema which elevates ICP which may cause serious herniations. Hyperosmolar agents, such as mannitol and hypertonic saline, are currently used to reduce elevated ICP and are recommended by the Brain Trauma Foundation. A less supported treatment route is decompressive craniectomy where a skull flap is removed to allow space for cerebral expansion. However, high mortality and complication rates make this option less beneficial and more controversial fo take place and aim to salvage neuronal damage which is repairable. Central to these acts include synaptic reorganization which alters the neuronal circuitry. The hippocampus and cortex are two regions particularly susceptible to damage and faulty repair in these regions contributes to abnormal electrical activity, resulting in seizures and possibly epilepsy. The BTF recommends the use of phenytoin for one week post-injury to reduce the risk for seizures. Other antiepileptic options are emerging, such as levetiracetam, as well as implantation of vagal nerve stimulators. Regardless, further research is warranted for the long-term use of anti-epileptic medications post-TBI (Figure 1).

Undoubtedly, the heterogeneous pathology of TBI makes uniform treatment recommendations difficult. A number of the reviewed studies recruited patients that were similar, whether it was in age, severity of injury, or imaging characteristics; narrowing the TBI population into homogenous groups yields clinically practical recommendations. Understanding the context, type of injury, and predominating pathophysiological mechanisms in a case will surely assist in the treatment and management of the TBI patient.

Figure 1 displays a general schematic regarding the overall pathophysiology of TBI. After injury, reduced CBF occurs from mechanical damage and leads to excitotoxicity-mediated cell death. Cell death produces an inflammatory state brought on by resident microglia and immune cells recruited from the periphery, leading to an elevation in ICP and a reduction in CPP. Inflammation eventually serves to repair the damage caused by TBI and is allows synaptic reorganization to occur. Reorganization and lasting damage increases susceptibility to seizures and possibly epilepsy. Hypothermia and HBOT target the deregulated cerebral metabolism and oxygen levels immediately after injury. Hyperosmolar agents, progesterone, and decompressive craniectomy seek to reduce inflammation caused by TBI and the ensuing damage. AEDs, such as LEV and PHT, and vagal nerve stimulation (VNS) reduce the probability of post-traumatic seizures.

## Figures and Tables

**Table 1.** Prophylactic Hypothermia Evidence in Traumatic Brain Injury (TBI). Table 1 summarizes results of evidence regarding the effect of prophylactic hypothermia on various outcomes. The selected results contain mixed results regarding the effectiveness of TBI.

Study	Design	Primary Outcome	Results	Notes
Bukur *et al*. 2012 * [45]	Retrospective	Spontaneous admission hypothermia on mortality	Pre-hospital hypothermia associated with increased mortality (Adjusted OR = 2.5)	95% CI = 1.1–6.3 *p* = 0.04 44 hypothermic patients 1790 normothermic patients
Rubiano *et al*. 2013 * [46]	Secondary analysis of Pennsylvania Trauma Outcome Study (PTOS)	Spontaneous admission hypothermia on mortality	Odds of death increased in spontaneous hypothermia group (OR = 1.70)	95% CI = 1.50–1.93 Odds adjusted for demographics, injury characteristics, and information at admission
Clifton *et al*. 2011 * [48]	Randomized controlled trial (RCT); National Acute Brain Injury Study: Hypothermia II (NABIS: H II)	Glasgow outcome scale (GOS) at 6 months post-injury	GOS nor mortality significantly differed between hypothermia and normothermia groups	Cooled to 33 °C for 48 h and rewarmed 0.5 °C every 2 h. Normothermia maintained at 37 °C
Suehiro *et al*. 2013 * [49]	Retrospective analysis of Japan Neurotrauma Data Bank Project (2009)	GOS	Hypothermia group had significantly more favorable outcomes compared with normothermia and no temperature management groups	Favorable outcomes—hypothermia (52.4%), normothermia (26.9%), No temperature management (20.7%)
Zhao *et al*. 2011 * [50]	Prospective randomized trial	GOS	Hypothermia group had improved outcome (75.0%) compared to normothermia (51.2%)	*p* = 0.038 Hypothermia maintained at 32.7 °C for 72 h. Spontaneous rewarming at room temperature. Normothermia maintained at 37 °C

* = Study as cited in text.

**Table 2.** Hyperbaric Oxygen Therapy Evidence in TBI. Table 2 summarizes varied evidence regarding the use of Hyperbaric Oxygen Therapy (HBOT) in the treatment of TBI and its effect on various primary outcomes. The selected evidence displays mixed results concerning the therapeutic benefit of HBOT.

Study	Design	Primary Outcome	Results	Notes
McDonagh *et al*. 2004 * [58]	Systematic review	Study outcome	Two studies demonstrated a benefit to HBOT. Five observational studies did not yield effective evidence	
Sahni *et al*. 2012 * [59]	Retrospective	Rancho Los Amigos Scale (RLAS)	Improved cognitive function in HBOT group (RLAS)	HBOT and standard treatment groups each had 20 patients
Lin *et al*. 2008 * [60]	Prospective randomized trial	GCS GOS	HBOT group had higher GCS improvement (*p* < 0.05); HBOT group had significant GOS improvement	HBOT and standard treatment groups each had 22 patients. GCS and GOS measured before HBOT and 3–6 months after
Rockswold *et al*. 2013 * [61]	Phase II RCT	Sliding dichotomized GOS and mortality	26% reduction in mortality (*p* = 0.0048) and 36% improvement in favorable GOS (*p* = 0.024) as compared to control	Treatment group given HBOT for 60 min at 1.5 atm followed by normobaric hyperoxia (3 h of 100% O_2_ at 1.0 atm)
Rockswold *et al*. 2010 * [62]	Prospective randomized trial	Metabolic markers (CSF lactate, cerebral metabolic rate of O_2_)	Reduced lactate and increased cerebral metabolic rate of O_2_ across both groups	Compared HBOT to normobaric hyperoxia (NBH)

* = Study as cited in text.

**Table 3.** Progesterone Administration Evidence in TBI. Table 3 summarizes presented evidence regarding the effect of progesterone administration on TBI outcomes, including GOS and mortality. Most of evidence presented favors the use of progesterone based on multiple different outcomes.

Study	Design	Primary Outcome	Results	Notes
Wright *et al*. 2007 * [115]	Phase II RCT with placebo	GOS-extended adverse events 30 day mortality	Progesterone and placebo group had similar adverse event rates. Progesterone had lower 30 day mortality. Moderate TBI patients receiving progesterone more likely to have improved outcome	Three days progesterone treatment 30 day mortality Rate ratio (RR) in progesterone group = 0.43 95% CI = 0.18–0.99 GOS-E in severe TBI patients; RR = 0.79; 95% CI = 0.29–2.13
Shakeri *et al*. 2013 * [116]	Prospective randomized trial	GOS	Significantly improved GOS and recovery in progesterone group (50%) compared to control (21%) at 3 months	1 mg/kg progesterone every 12 h for 5 days; Patients with GCS ≤ 8 enrolled
Ma *et al*. 2012 * [117]	Meta-analysis	Mortality	Progesterone reduced mortality at end of follow-up and disability	Mortality with progesterone pooled risk ratio = 0.61, 95% CI = 0.40–0.93 Death and severe disability with progesterone pooled risk ratio = 0.77, 95% CI = 0.62–0.96
Aminmansour *et al*. 2012 * [118]	RCT with placebo	GOS	Favorable Outcomes at 3 months Placebo = 25% Progesterone = 45% Progesterone + Vitamin D = 60% *p* = 0.03	Separate group receiving progesterone and vitamin D included

* = Study as cited in text.

**Table 4.** Hyperosmolar Agents Evidence in TBI. Table 4 summarizes selected evidence regarding hyperosmolar agents in TBI. The evidence compares the use of hypertonic saline (HS) and mannitol in reducing ICP during neuroinflammation. Mannitol is the gold standard hyperosmolar agent recommended for use by the BTF. However, the evidence provides strong support in favor of HS.

Study	Design	Primary Outcome	Results	Notes
Rickard *et al*. 2013 * [121]	Meta-analysis	Pooled mean ICP reduction	Weighted mean ICP reduction difference with hypertonic saline compared mannitol = 1.39 mmHg, 95% CI = −0.74–3.53	Six studies with 171 patients and 599 episodes of raised ICP included
Ichai *et al*. 2013 * [122]	RCT receving either half-molar sodium lactate (SL) or isotonic saline	Raised ICP episodes (≥20 mmHg)	Half-molar SL group had significantly fewer raised ICP episodes compared to control (*p* < 0.05)	Patients received 48 h continuous infusion (0.5 mL/kg/h)
Eskandari *et al*. 2013 * [123]	Prospective cohort study	Refractory intracranial hypertension treatment response	Boluses significantly decreased ICP and sustained the decrease and elevated CPP	Using 14.6% Hypertonic Saline Boluses repeated every 15 min. over 12 h
Oddo *et al*. 2009 * [124]	Prospective study	Elevated ICP refractory to mannitol—Response to hypertonic saline (HS)	HS significantly elevated brain tissue oxygenation, reduced ICP, and elevated cardiac output compared to mannitol	7.5%, 250 mL HS treatment
Cottenceau *et al*. 2011 * [125]	Randomized prospective study	ICP, CPP, CBF, outcome	Mannitol and HS both reduced ICP and elevated CPP and CBF. HS had significantly more pronounced effect over greater duration. No difference in outcome between two groups	20% Mannitol (4 mL/kg) 7.5% HTS (2 mL/kg)

* = Study as cited in text.

**Table 5.** Anti-Epileptic Drugs Evidence in TBI: Case for Levetiracetam. Table 5 displays selected evidence regarding the use of levetiracetam (LEV) in preventing seizures post-TBI, often as compared to phenytoin (PHT). PHT is the mainstay treatment for seizure prophylaxis as recommended by the BTF. Many studies display similar results on seizure prevention concerning LEV and PHT. However, LEV may be more clinically practical than PHT.

Study	Design	Primary Outcome	Results	Notes
Pearl *et al*. 2013 * [168]	Phase II prospective trial	Posttraumatic epilepsy (PTE) development sdverse events mortality	1/40 patients developed PTE. Non-serious adverse events: headache, fatigue, irritability, drowsiness. No mortality at follow-up	Children 6–17 years LEV 55 mg/kg/day for 30 day. 2 year follow-up
Zafar *et al*. 2012 * [169]	Meta-analysis	seizures between LEV and PHT	Neither drug was superior to the other in reducing seizures	Pooled OR = 0.96 95% CI = 0.24–3.79
Kruer *et al*. 2013 * [171]	retrospective observational study	posttraumatic seizures	89 received PHT, 20 received LEV. 1 patient suffered a seizure in each group	Most patients had AED prophylaxis for >7 days despite guidelines
Szaflarski *et al*. 2010 * [172]	Prospective, randomized, single-blind trial PHT *vs*. LEV	GOS disability rating scale (DRS) seizures mortality	LEV had lower DRS (*p* = 0.042) and higher GOS (*p* = 0.039). No significant difference in seizure occurrence or mortality.	DRS completed at 3 months, GOS at 6 months. Continuous EEG used to measure seizure occurrence over initial 72 h.
Inaba *et al*. 2013 * [173]	Prospective study PHT *vs*. LEV	Early post-traumatic seizures mortality	No difference between LEV and PHT in seizure rate (1.5% *vs*. 1.5%, *p* = 0.997) or mortality (5.4% *vs*. 3.7%, *p* = 0.236)	LEV: 1,000 mg every 12 h; PHT: loading dose 20 mg/kg, maintenance dose 5 mg/kg/d rounded to nearest 100 mg.

* = Study as cited in text.
